# Genome-Wide CRISPR Screening Identifies JAK1 Deficiency as a Mechanism of T-Cell Resistance

**DOI:** 10.3389/fimmu.2019.00251

**Published:** 2019-02-19

**Authors:** Ping Han, Qiang Dai, Lilv Fan, Hao Lin, Xiaoqing Zhang, Fanlin Li, Xuanming Yang

**Affiliations:** School of Life Sciences and Biotechnology, Shanghai Jiao Tong University, Shanghai, China

**Keywords:** JAK1 deficiency, CRISPR/Cas9, T-cell resistance, immunotherapy, melanoma

## Abstract

Somatic gene mutations play a critical role in immune evasion by tumors. However, there is limited information on genes that confer immunotherapy resistance in melanoma. To answer this question, we established a whole-genome knockout B16/ovalbumin cell line by clustered regularly interspaced short palindromic repeats (CRISPR)/CRISPR-associated protein-9 nuclease technology, and determined by *in vivo* adoptive OT-I T-cell transfer and an *in vitro* OT-I T-cell-killing assay that Janus kinase (JAK)1 deficiency mediates T-cell resistance via a two-step mechanism. Loss of JAK1 reduced JAK-Signal transducer and activator of transcription signaling in tumor cells—resulting in tumor resistance to the T-cell effector molecule interferon—and suppressed T-cell activation by impairing antigen presentation. These findings provide a novel method for exploring immunotherapy resistance in cancer and identify JAK1 as potential therapeutic target for melanoma treatment.

## Introduction

Melanoma is a common malignant form of skin cancer; once metastasis has occurred, the prognosis is unfavorable. In recent years, the incidence of melanoma has increased markedly worldwide including in China, whereas in Europe and United States the mortality rate has been relatively stable. There is a need for more effective treatments for melanoma. Although immune-based therapies for metastatic melanoma involving cytokines, adoptive T-cell transfer, and checkpoint blockade have achieved some success (1–4), delayed relapse often occurs after initial tumor arrest or regression even with continuous therapy. For example, about 25% patients with melanoma who received Programmed cell death protein (PD)-1 blockade therapy and were initially responsive but developed resistance after about 21 months (5).

Gene mutations can induce the production of neoantigens that elicit potent T-cell responses (6–8). Recent evidence suggests that somatic gene mutations in tumors also induce immune resistance to immunotherapies, especially those that block immune checkpoint molecules such as cytotoxic T-lymphocyte-associated antigen (CTLA)-4 or PD-1 (9, 10). Gene mutations are detected in most cancers including melanoma (11), which has a particularly high mutation load (12)—for instance, mutations in the mitogen-activated protein kinase, phosphatidylinositol 3-kinase/AKT, and HIPPO signaling pathways were shown to mediate immune resistance (13–16). However, most of the above studies screened components of a single pathway to identify gene mutations conferring immune resistance; as such, there are likely many additional mutations that remain to be identified.

Clustered regularly interspaced short palindromic repeats (CRISPR) is a microbial nuclease system that contains a combination of CRISPR/CRISPR-associated protein-9 nuclease (Cas) genes as well as non-coding RNA elements that dictate the specificity of CRISPR-mediated nucleic acid cleavage. A type II CRISPR nuclease system targeting early consecutive exons and delivered by lentivirus achieved genome editing in mammalian cells, generating a pooled library (17); and a genomic CRISPR knockout (KO) library has been used to screen the loss of function effects of genes associated with cancer cell proliferation (18, 19), drug resistance (17, 20), metastasis (21), and tumor immunity (22, 23).

Janus kinase (JAK)1 mutation is linked to immune resistance in multiple tumor types including endometrial, colorectal, stomach, and prostate carcinomas (24). In some cancers such as fibrosarcoma and acute lymphoblastic lymphoma, JAK1 mutations activate JAK–Signal transducer and activator of transcription (STAT)–interleukin-6 signaling to promote tumor cell proliferation (25, 26) whereas in most other cancers, JAK1 mutations impair interferon (IFN) signaling and induce immune resistance (27–29). Loss of JAK1 was shown to stimulate human adenocarcinoma cells unresponsive to IFN (30), suggesting that immune resistance induced by JAK1 deficiency is related to IFN signaling. Indeed, mutations in components of this pathway including JAK1, JAK2, β2 microglobulin (β2M), and IFN-γ receptor (IFNGR)1 caused resistance to antibodies against the immune checkpoint molecules Programmed death ligand (PD-L)1 or CTLA-4 (31–33). In addition, IFN-γ-resistant human melanoma cells with JAK1 or JAK2 mutation were resistant to the cytotoxic effects of T-cells (34). These studies demonstrate that JAK1 in IFN signaling plays an essential role in resistance to T-cell-based immunotherapies through mechanisms that remain unclear.

Here we used a mouse genome-scale CRISPR (GeC)KO pooled library to screen all potential gene mutations mediating T-cell resistance and identified JAK1 loss-of-function as a major contributor to this process.

## Materials and Methods

### Mice

OT-I cluster of differentiation (CD)8^+^ T-cell receptor transgenic and IFN-α receptor (IFNAR) KO (IFNAR^−/−^) mice were purchased from The Jackson Laboratory (Bar Harbor, ME, USA). C57BL/6J mice were from Beijing Vital River Laboratory Animal Technology Co. (Beijing, China). The mice were maintained under specific pathogen-free conditions. Animal care and use were carried out in accordance with institutional and National Institutes of Health protocols and guidelines, and all studies were approved by the Animal Care and Use Committee of Shanghai Jiao Tong University.

### Cell Lines and Reagents

B16/ovalbumin (OVA) cells were provided by Hans Schreiber (University of Chicago). B16/OVA-JAK1^−/−^ cells were generated using CRISPR/Cas9 technology. Briefly, B16/OVA cells were infected with lentivirus expressing Cas9 and single guide (sg)RNA against mouse JAK1. After puromycin selection, resistant cells were subcloned and *JAK1* and mutant (mut)*JAK1* were amplified from C57BL/6J mouse total DNA by overlap PCR and cloned into the pMSCV vector. B16/OVA-JAK1^−/−^ cells were infected with lentivirus expressing mutJAK1 and selected with blasticidin to obtain B16/OVA-JAK1^−/−^-mutJAK1. B16/OVA cells and their derivatives were cultured at 37°C under 5% CO_2_ in Dulbecco's Modified Eagle's Medium supplemented with 10% heat-inactivated fetal bovine serum (FBS; Gibco, Grand Island, NY, USA), 2 mmol/L l-glutamine, 100 U/mL penicillin, and 100 μg/mL streptomycin. Anti-mPD-L1 (10F.9G2) antibody was purchased from Bio X Cell (West Lebanon, NH, USA).

### Screening of Genes Related to T-Cell Resistance

The genome-scale KO B16/OVA cell line was transduced with the lentiviral mouse CRISPR KO (GeCKO) v2.0 pooled library (35). For *in vitro* experiments, GeCKO-B16/OVA cell lines were stimulated with OT-I peptide at a concentration of 0.2 μg/mL for 0.5 h, then cocultured with OT-I T-cells (E: T = 3:1). For *in vivo* experiments, GeCKO-B16/OVA cell lines and OT-I T-cells were subcutaneously injected (E: T = 1:1) into the right flank of 7- to 9-week-old mice; 1 month later, the mice were sacrificed, their tumors removed and digested, and the dissociated tumor cells were cultured. Immune-resistant cells obtained from *in vitro* and *in vivo* experiments were co-cultured with OT-I T-cells (E: T = 3:1) separately; after two rounds of killing by OT-I T-cells, RNA was isolated from the remaining immune-resistant cells and sgRNA was obtained by reverse transcription PCR. Next-generation sequencing (NGS) of sgRNAs was performed by Genewiz (Suzhou, China).

### Tumor Inoculation and Treatments

Approximately 10^6^ B16/OVA cells or their derivatives were subcutaneously injected into the right flank of 7- to 9-week-old mice. Tumor volume was measured and calculated as V = (W^2^ × L)/2 (36). After tumors were established (~8–12 days), mice were subjected to three intratumoral injections of OT-I T-cells or anti-mouse PD-L1 or IFN-α or control antibody every 4 days.

### Detection of IFN-γ Secretion by Cytometric Bead Array (CBA)

Antitumor-specific T-cells were detected with the CBA assay. Spleen or lymph node (LN) cells were resuspended in Roswell Park Memorial Institute 1640 supplemented with 10% FBS, 2 mmol/L l-glutamine, 100 U/mL penicillin, and 100 μg/mL streptomycin. A total of 1–2 × 10^5^ cells were used for the assay. Irradiated tumor cells were added at a 1:3 ratio to spleen or LN cells; after 48 h of incubation, IFN-γ level was determined with the IFN-γ CBA assay (BD Biosciences, Franklin Lakes, NJ, USA).

### Flow Cytometry

Single-cell suspensions were incubated with anti-CD16/32 antibody (anti-FcJIII/II receptor, clone 2.4G2) for 10 min and then labeled with fluorophore-conjugated antibody (BioLegend, San Diego, CA, USA or eBioscience, San Diego, CA, USA). Samples were analyzed by flow cytometry (Cytoflex; Beckman Coulter, Brea, CA, USA or Sony Biotech, San Jose, CA, USA), and data were analyzed with FlowJo software (Tree Star, Ashland, OR, USA).

### Statistical Analysis

Data are expressed as mean ± SEM and were compared with the two-tailed unpaired Student's *t*-test. *P* < 0.05 was considered statistically significant.

## Results

### Identification of JAK1 as a Candidate Molecule Inducing T-Cell Resistance by Genome-Wide CRISPR/Cas9 Screening

**Figure 1:**
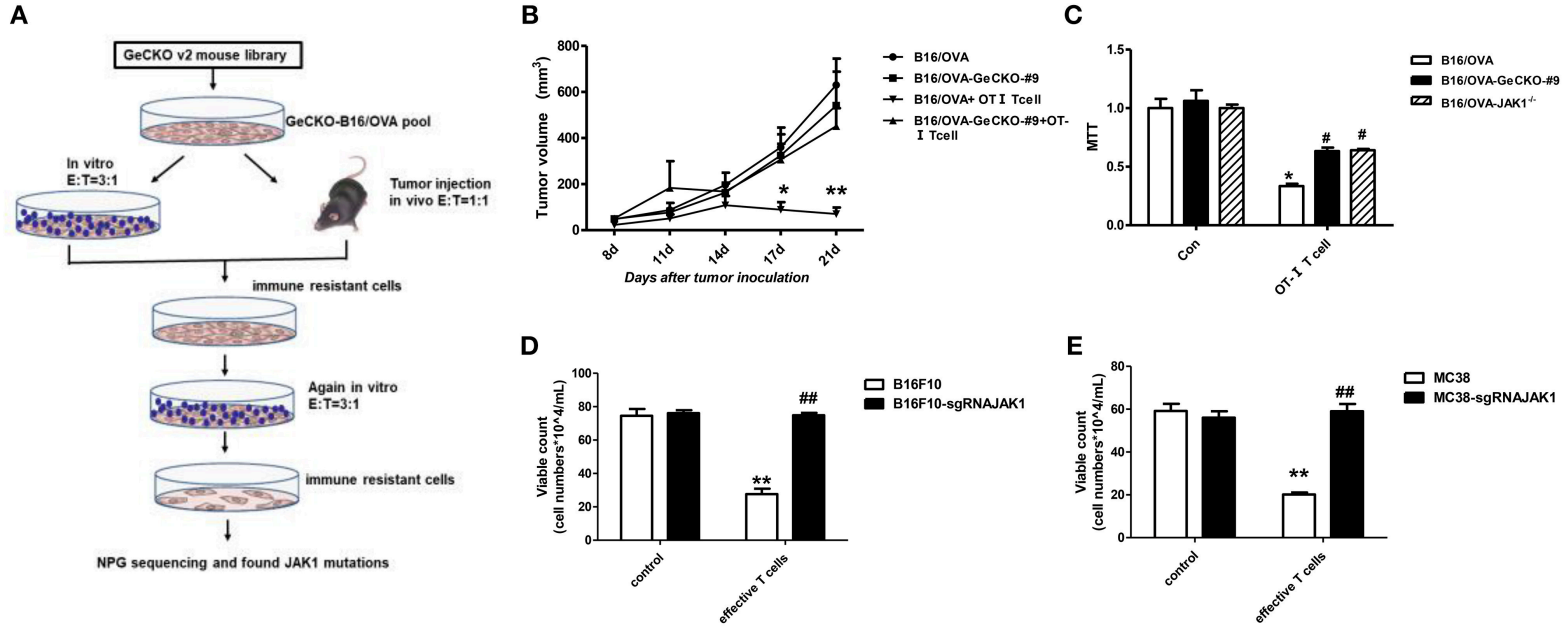
Identification of JAK1 as a candidate factor responsible for T-cell resistance by genome-wide CRISPR/Cas9 screening. **(A)** Screening procedure for genes associated with B16/OVA resistance to T-cell killing. **(B)** Wild-type (WT) B6 mice (*n* = 5/group) were subcutaneously injected with 5 × 10^5^ B16/OVA or B16/OVA-GeCKO#9 cells, then 1 × 10^6^ OT-I T-cells were administered on days 8 and 12. Tumor size was measured twice a week. Data represent mean ± SEM. **(C)** B16/OVA, B16/OVA-GeCKO#9, and B16/OVA-JAK1^−/−^ were co-cultured with OT-I T-cells and tumor cell viability was evaluated 72 h later. **(D,E)** Number of live JAK1-deficient B16F10 and MC38 cells co-cultured with specific T-cells for 72 h. Data are presented as mean ± SEM. ^*^P < 0.05 vs. B16/OVA/B16F10/MC38 control group; ^#^*P* < 0.05 vs. B16/OVA/B16F10/MC38^+^ T-cell group. ^**^P < 0.01 vs. B16/OVA/B16F10/MC38 control group; ^##^P < 0.01 vs. B16/OVA/B16F10/MC38+ T-cell group.

We established a stable B16/OVA cell line (GeCKO B16/OVA) by infection with mouse GeCKO lentiviral library containing 130,209 unique sgRNAs targeting 20,611 genes. In this cell line, OVA is stably expressed and serves as a tumor-specific model antigen. Peptides OT-I and -II generated from OVA can be presented by major histocompatibility complex (MHC)-I and -II, respectively, to activate OT-I- and -II-specific T-cell responses. Since CD8^+^ T-cells are the most important mediators of anti-tumor immunity, we used tumor-specific OT-I T-cells (reactive to OVA peptide) to screen GeCKO B16/OVA cells in order to identify candidate genes involved in immune resistance. After two rounds of screening both *in vitro* and *in vivo*, remaining cells (B16/OVA-GeCKO-#9) were considered as immune-resistant tumor cells. An analysis of the inserted sgRNA by NGS sequencing revealed a JAK1-targeting sgRNA (TAGAAAGTCACCTCCACTCC) in almost 60–70% of samples (Figure 1A). To confirm that JAK1 deficiency leads to T-cell resistance, T-cell killing experiments were performed both *in vitro* and *in vivo*. Compared to the B16/OVA + OT-I T-cell group, the B16/OVA-GeCKO-#9 + OT-I T-cell group was resistant to the cytotoxic effects of T-cells (Figure 1B). To exclude the possibility that lentivius-mediated random insertion activated the endogenous gene to indirectly induce resistance, we reconstructed the B16/OVA-JAK1^−/−^ cell line using the CRISPR/Cas9 method. The results of the *in vitro* OT-I T-cell killing assay showed that B16/OVA-JAK1^−/−^ cells exhibited a T-cell-resistant phenotype similar to B16/OVA-GeCKO-#9 (Figures 1B,C). To verify the generalizability of these findings, we performed similar experiments with B16F10-sgRNAJAK1 and MC38-sgRNAJAK1 tumor cell lines generated by CRISPR/Cas9 method. Irradiated B16F10 and MC38 cell lines were used to vaccinate B6 mice three times to stimulate the production of B16F10 or MC38 specific T-cells; sensitivity to T-cells specific to B16F10-sgRNAJAK1 or MC38-sgRNAJAK1 was confirmed (Figures 1D,E). These results suggest that JAK1 loss or mutation contributes to tumor resistance to immunotherapies.

### Loss of JAK1 Attenuates JAK–STAT Signaling in Tumor Cells

**Figure 2:**
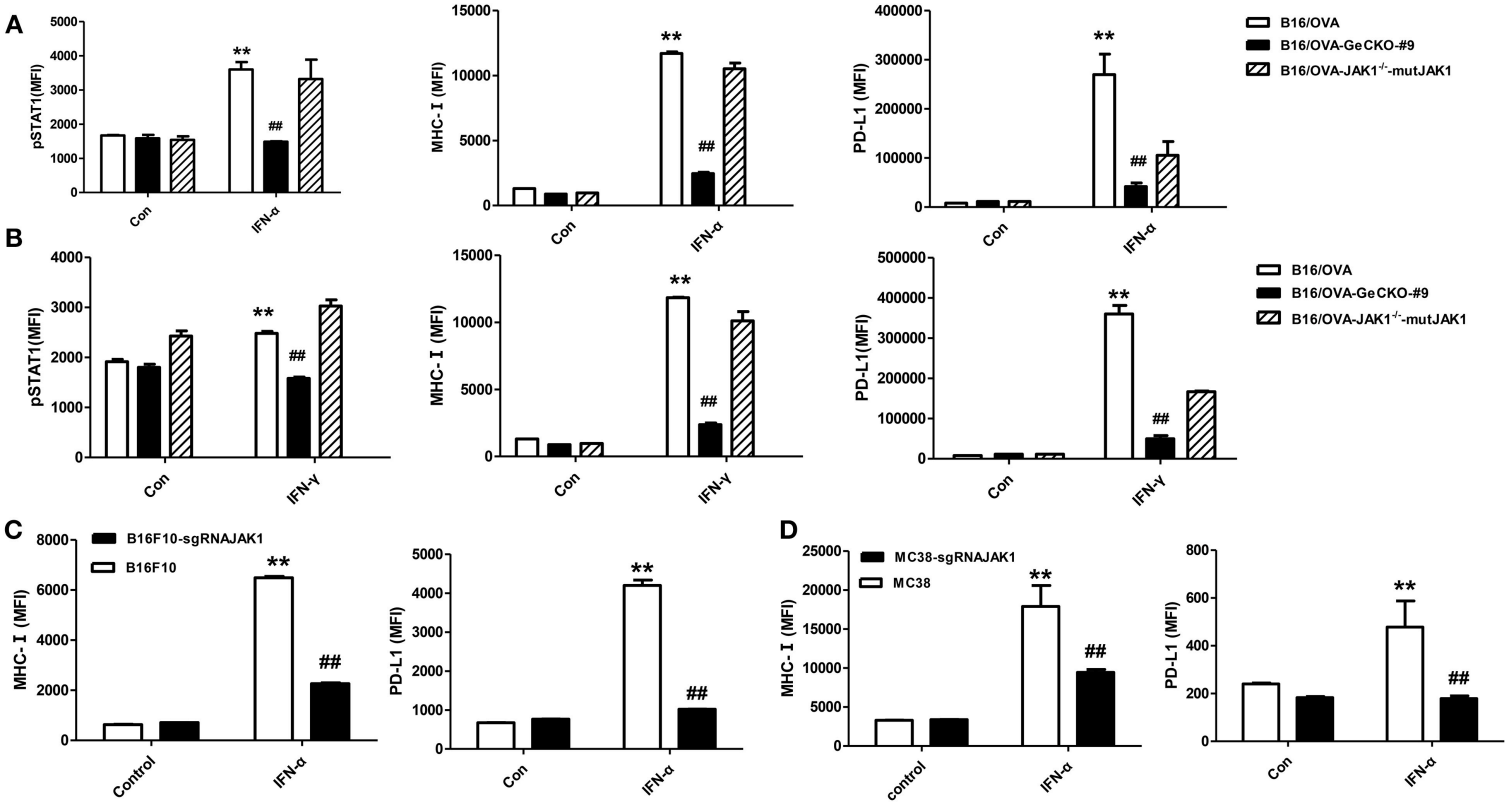
Loss of JAK1 attenuates JAK–STAT signaling in tumor cells. **(A,B)** B16/OVA, B16/OVA-JAK1^−/−^, and B16/OVA-JAK1^−/−^-mutJAK1 were stimulated with mouse (m)IFN-γ (100 ng/mL) **(A)** or mIFN-α4 (500 ng/mL) **(B)**, and pSTAT1 level was analyzed by flow cytometery 0.5 h later. MHC-I and PD-L1 expression was analyzed by flow cytometry 24 h after stimulation. MHC-I and PD-L1 expression in B16F10 and MC38 cells with JAK1 deficiency were observed after stimulation with mIFN-α4 (500 ng/mL) **(C,D)**. Data are presented as mean ± SEM. ^**^P < 0.01 vs. B16- OVA/B16F10/MC38 control group; ^##^*P* < 0.01 vs. B16/OVA/B16F10/MC38 + IFN-α group.

JAK1 is the key downstream kinase in type I and II IFN signaling. Besides directly inducing tumor cell apoptosis, type I IFNs and IFN-γ promote anti-tumor immunity via multiple mechanisms—i.e., directly activating T-cells and activating natural killer cells along with dendritic cells to cross-prime CD8 T-cells. Since JAK1 is a critical kinase for STAT phosphorylation, we examined the level of phosphorylated (p)STAT1 following IFN stimulation. After either type I IFN (IFN-α4) or IFN-γ stimulation (Figures 2A,B), STAT1 phosphorylation was much lower in B16/OVA cells lacking JAK1 than in those with normal expression. We also observed a downregulation of MHC-I and PD-L1, which have been previously reported as IFN-inducing genes. To test whether JAK1 is directly responsible for impaired IFN signaling, we re-expressed codon-optimized JAK1 in B16/OVA-JAK1^−/−^ (B16/OVA-JAK1^−/−^-mutJAK1) cells, which are resistant to sgRNA-mediated gene editing; this restored the expression pSTAT1, MHC-I, and PD-L1, suggesting that loss of JAK1 directly impairs the activation of the IFN pathway. Downregulation of MHC-I and PD-L1 were also observed in B16F10 and MC38 cell lines lacking JAK1 in response to IFN-α4 stimulation (Figures 2C,D).

### Loss of JAK1 Compromises Tumor-Specific T-Cell Activation

**Figure 3:**
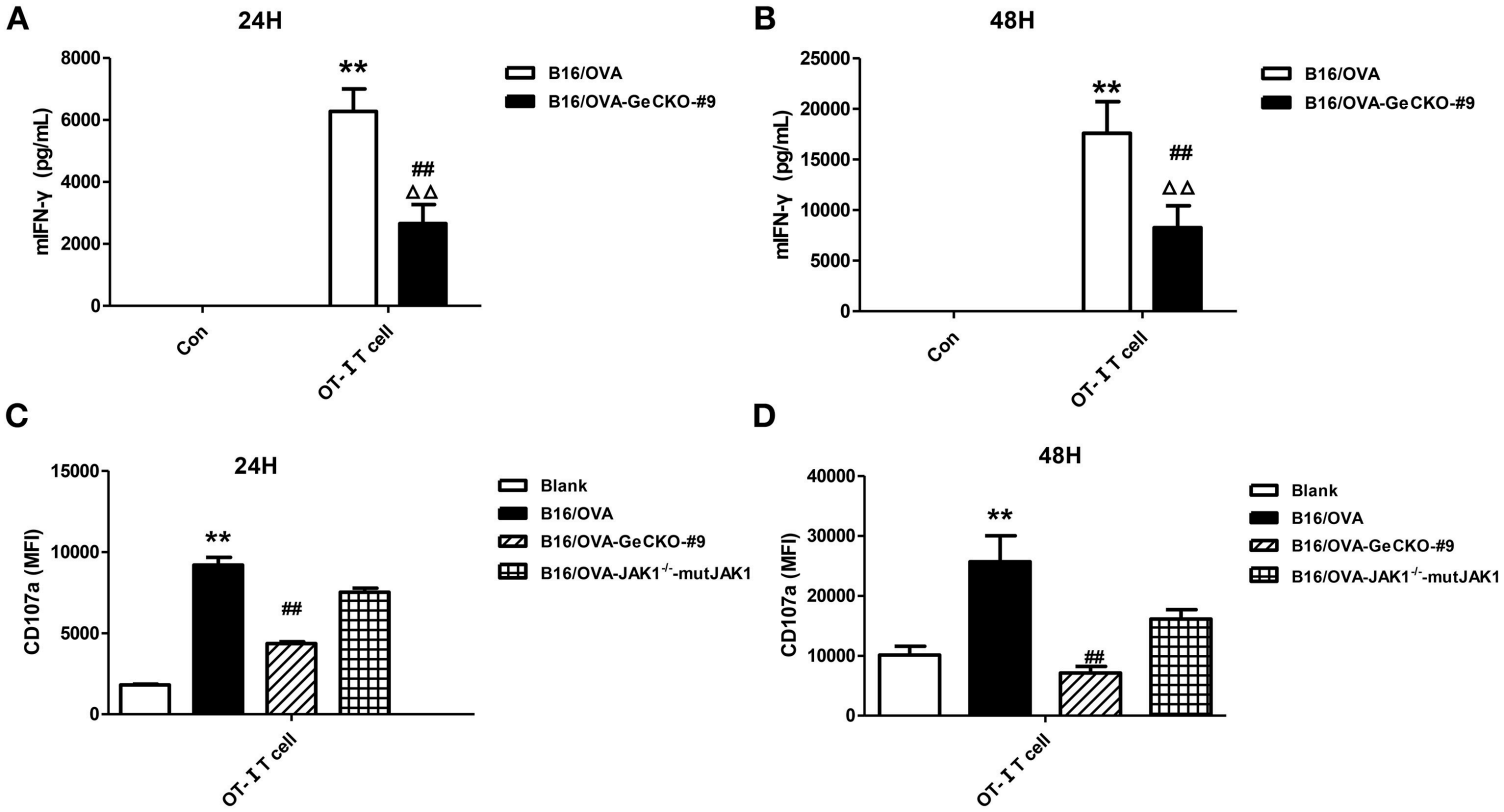
Loss of JAK1 reduces tumor-specific T-cell activation. OT-I T-cells and indicated B16/OVA cells and their derivatives were co-cultured (E: T = 3:1) for 48 h, and IFN-γ level in the supernatant **(A,B)** and CD107a expression in OT-I T-cells **(C,D)** were measured. Data are presented as mean ± SEM. ^**^P < 0.01 vs. B16-OVA control group; ^##^*P* < 0.01 vs. B16/OVA + OT-I T-cell group; ^ΔΔ^*P* < 0.01 vs. B16/OVA-GeCKO-#9 control group.

Based on the observation that B16/OVA cells lacking JAK1 are resistant to OT-I T-cell killing we speculated that B16/OVA-JAK1^−/−^ cells cannot activate OT-I T-cells or that they activate OT-I T-cells but are resistant to the toxic effects of effector molecules released by T-cells. To evaluate the first possibility, we used B16/OVA and B16/OVA-JAK1^−/−^ cells to stimulate OT-I T-cells and examined T-cell activation status, with expression of two effector molecules serving as a readout of IFN-γ secretion and CD107a degranulation. We found that OT-I T-cells stimulated by B16/OVA cells lacking JAK1 secreted less IFN-γ and expressed less CD107a. Furthermore, when we re-expressed codon-optimized JAK1 in B16/OVA-JAK1^−/−^ cells, IFN-γ and CD107a levels in B16/OVA-JAK1^−/−^-mutJAK1 cells were restored (Figure 3). Thus, JAK1 deficiency impairs the activation of tumor-specific T-cell responses.

### Loss of JAK1 Reduces the Cytotoxicity of T-Cells

**Figure 4:**
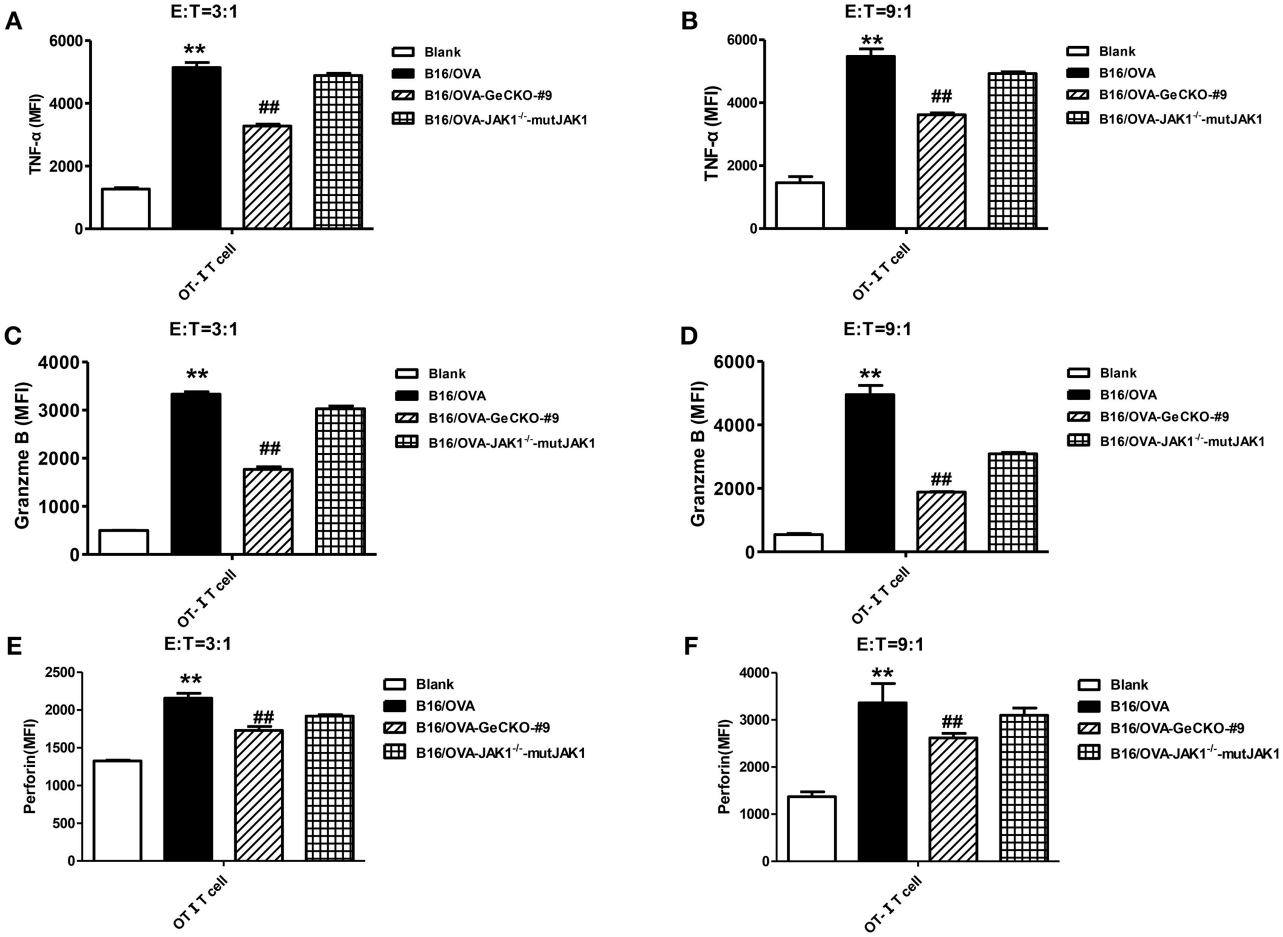
Loss of JAK1 decreases the cytotoxicity of T-cells. OT-I T-cells and indicated B16/OVA cells and their derivatives were co-cultured (E: T = 3:1 or 9:1) for 48 h, and the production of TNF-α **(A,B)**, granzyme B **(C,D)**, and perforin **(E,F)** were analyzed by intracellular flow cytometry. Data are presented as mean ± SEM. ^**^P < 0.01 vs. blank group; ^##^*P* < 0.01 vs. B16/OVA + OT-I T-cell group.

To further investigate whether the absence of JAK1 affects T-cell cytotoxicity, tumor-specific OT-I T-cells were co-cultured with B16/OVA or B16/OVA-JAK1^−/−^ cells and the expression of the cytotoxicity-related effector molecules tumor necrosis factor (TNF)-α, granzyme B, and perforin was analyzed. TNF-α (Figures 4A,B), granzyme B (Figures 4C,D), and perforin (Figures 4E,F) were all downregulated in cells lacking JAK1; this was rescued by the re-expression of codon-optimized JAK1 (Figure 4), implying that JAK1 deficiency prevents killing by tumor-specific T-cells.

### Loss of JAK1 Inhibits Tumor Cell Apoptosis

**Figure 5:**
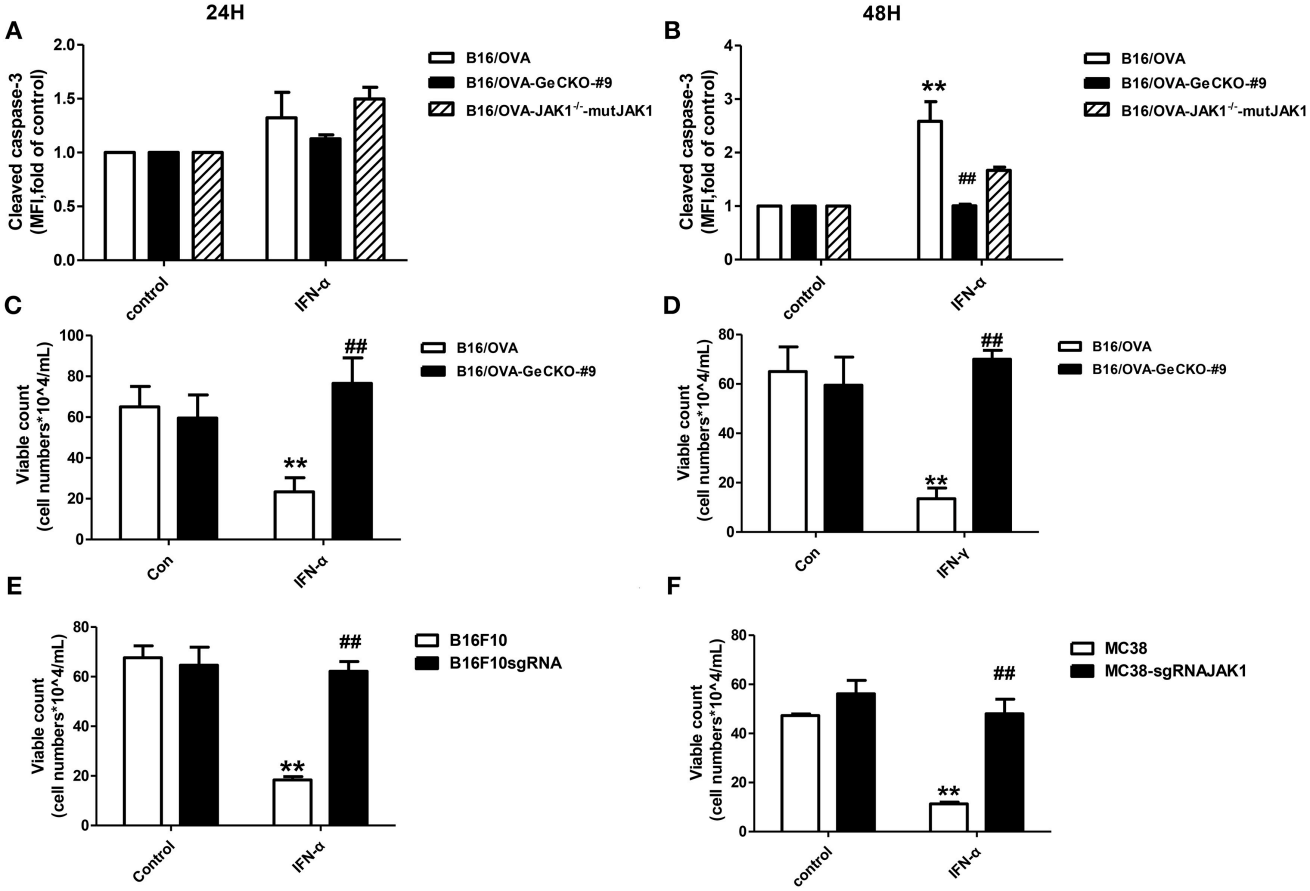
Loss of JAK1 inhibits tumor cell apoptosis. B16/OVA cells and their derivatives were stimulated with mIFN-γ (100 ng/mL) or mIFN-α4 (500 ng/mL) for 72 h. **(A,B)** Cleaved caspase-3 expression was analyzed by flow cytometry 24 h later. **(C,D)** Remaining live cells were counted with a hemocytometer after 72 h. **(E,F)** B16F10 and MC38 with JAK1 were stimulated with mIFN-α4 (500 ng/mL) for 72 h and the remaining live cells were counted. Data are presented as mean ± SEM. ^**^P < 0.01 vs. B16/OVA/B16F10/MC38 control group; ^##^*P* < 0.01 vs. B16/OVA/B16F10/MC38 + IFN-α group.

We showed that loss of JAK1 impairs JAK–STAT signaling and negatively regulates T-cell activation and function. It is known that the JAK–STAT pathway regulates multiple apoptosis-related factors including nuclear factor (NF)-κB, B cell lymphoma (Bcl)-2/Bcl-2-associated X protein (Bax), and Suppressor of cytokine signaling (SOCS). We therefore examined whether loss of JAK1 affects the apoptosis of B16/OVA tumor cells. IFN-α4 was used to induce apoptosis of B16/OVA and B16/OVA-JAK1^−/−^ cells, and cleaved caspase-3 activation was analyzed by flow cytometry. Compared to B16/OVA cells expressing JAK1, caspase-3 level was reduced in IFN-α-treated B16/OVA-JAK1^−/−^ cells. JAK1 re-expression in B16/OVA-JAK1^−/−^ cells restored this expression (Figures 5A,B). Consistent with these observations, there were more live B16/OVA-JAK1^−/−^ cells after IFN-α4 or IFN-γ treatment (Figures 5C,D) and live B16F10 and MC38 cells lacking JAK1 (Figures 5E,F). These data suggest that loss of JAK1 inhibits the apoptosis of tumor cells stimulated by type I or II IFNs.

### JAK–STAT Signaling Plays Distinct Roles in Immunotherapy of Tumor and Non-tumor Cells

**Figure 6:**
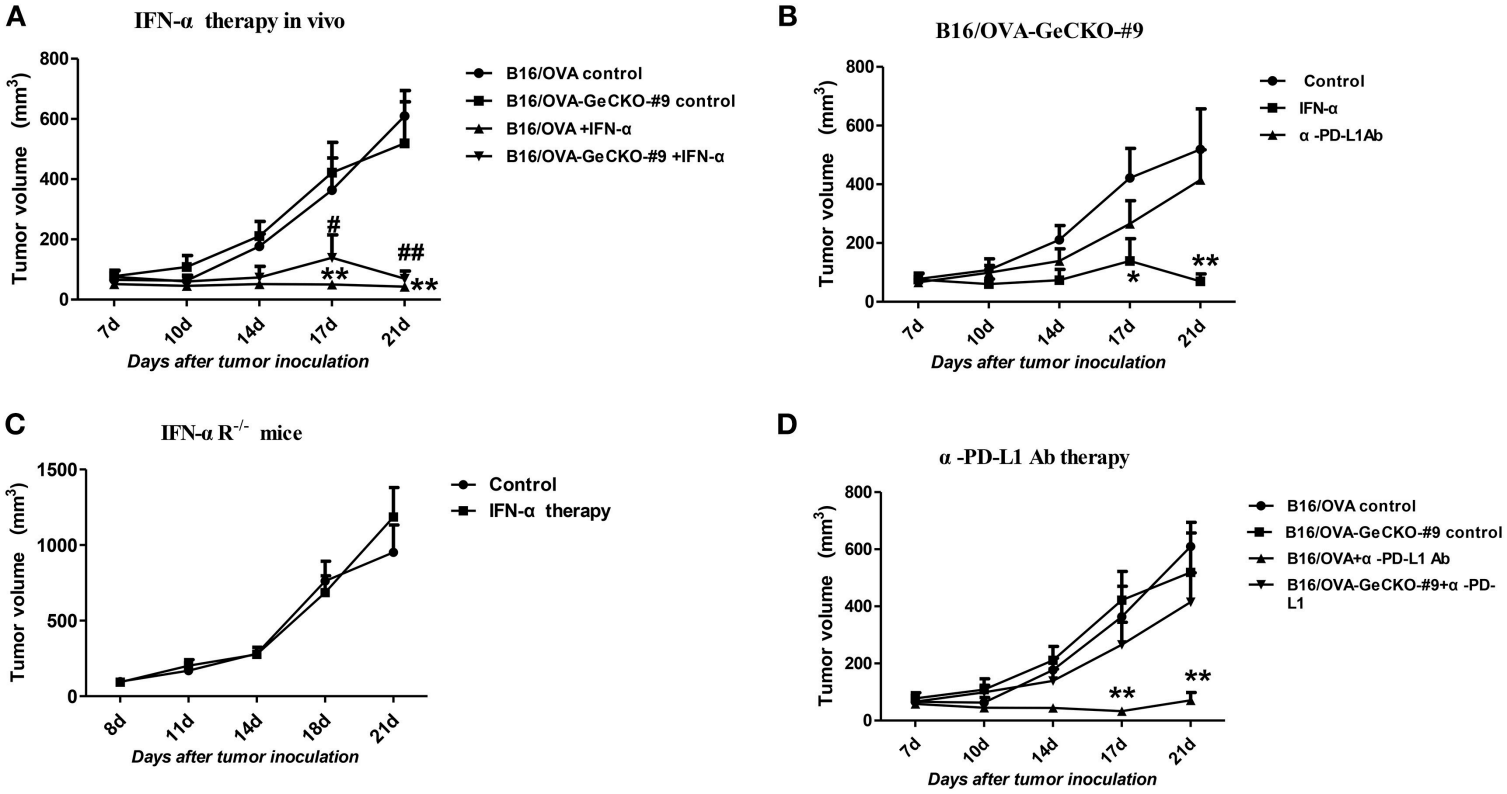
JAK–STAT signaling is differentially activated in tumor and non-tumor cells in response to various immunotherapies. **(A–D)** Wild-type (WT) B6 or IFNAR1^−/−^ mice (*n* = 5/group) were subcutaneously injected with 5 × 10^5^ B16/OVA cells and their derivatives; starting 8 days later, tumors were injected with 5 μg IFN-α **(A–C)** or 25 μg anti-PD-L1 antibody **(D)** or control antibody twice a week. Tumor volume was also measured twice a week. Data are presented as the mean ± SEM. ^*^P < 0.05 vs. B16-OVA GeCKO-#9 control group; ^**^P < 0.01 vs. B16-OVA GeCKO-#9 control group **(B)**; ^**^P < 0.01 vs. B16-OVA control group; ^#^*P* < 0.05 vs. B16/OVA-GeCKO-#9 control group; ^##^P < 0.01 vs. B16/OVA-GeCKO-#9 control group; **(A,D)**.

Since loss of JAK1 induced tumor resistance to IFNs *in vitro*, we examined whether it altered the therapeutic effects of IFN-α *in vivo* by treating mice bearing B16/OVA or B16/OVA-JAK1^−/−^ cell-derived tumors with IFN-α4. Unexpectedly, IFN-α treatment suppressed the growth of B16/OVA and B16/OVA-JAK1^−/−^ cell-derived tumors (Figures 6A,B), suggesting that type I IFN signaling in the host contributes to the anti-tumor efficacy of IFN-α4. To test this hypothesis, mice with B16/OVA-JAK1^−/−^ cell-derived tumors were treated with IFN-α4. Consistent with our hypothesis, the therapeutic effect of IFN-α4 was markedly reduced in IFNAR1^−/−^ mice, implying that JAK–STAT signaling in tumor and non-tumor cells is important for the anti-tumor effects of IFN-α4 (Figure 6C). To determine whether loss of JAK1 influences the therapeutic effects of immune checkpoint blockade, mice bearing B16/OVA or B16/OVA-JAK1^−/−^ cell-derived tumors were treated with anti-PD-L1 antibody (Figure 6D). We found that the therapeutic effect of the antibody was diminished in this tumor model. These data indicate that JAK–STAT signaling is equally important in tumor and non-tumor cells but has distinct mechanisms of action in the two cell types.

## Discussion

**Figure 7:**
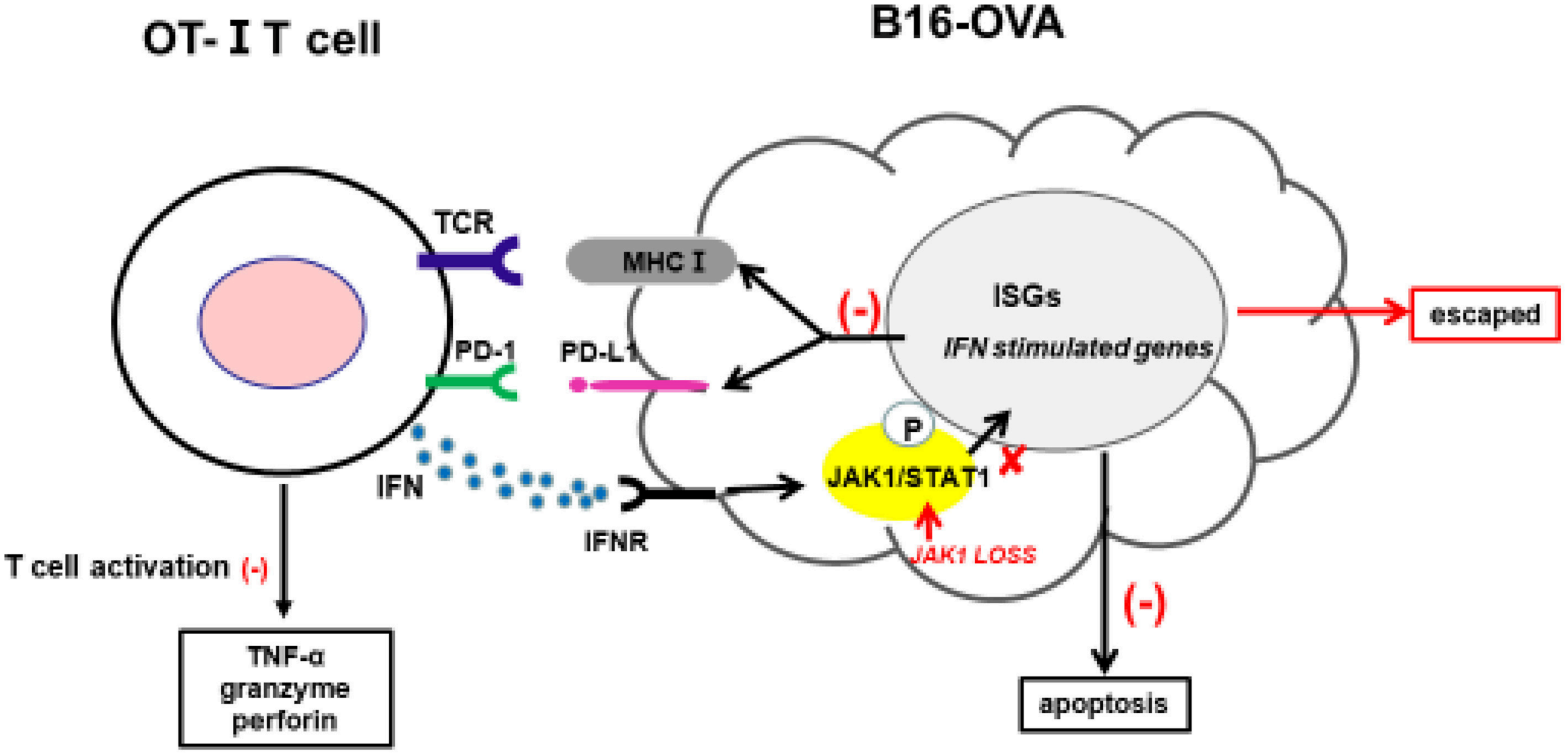
Proposed mechanism by which JAK1 deficiency leads to cytotoxic T-cell resistance. Tumor-derived OT-I–MHC-I complex can activate OT-I T-cells to produce cytotoxic IFN-γ, TNF-α, granzyme, and perforin. IFN-γ can activate JAK–STAT signaling in tumor cells, leading to upregulation of multiple regulatory genes affecting T-cell activation and function. IFN-γ-induced MHC-I can sensitize tumor cells to T-cell-mediated killing, while IFN-γ-induced PD-L1 suppresses T-cell activation by interacting with PD-1 in T-cells.

Cancer immunotherapy—including immune checkpoint antibodies and chimeric antigen receptor T-cell therapies—has achieved clinical success; however, a fraction of patients exhibit resistance. Clarifying the underlying mechanisms is critical for overcoming this resistance and developing more effective cancer treatments. The intrinsic resistance mechanism of tumors to adoptive T-cell transfer is not well-understood; in the present study, we used a novel CRISPR/Cas9-based whole-genome screening strategy to identify key mutations that are involved. Our results demonstrate that: 1) loss of JAK1 induces T-cell resistance in tumor cells *in vitro* and *in vivo*; 2) both type I and II IFN pathways are impaired in the absence of JAK; 3) JAK1-deficient tumor cells are resistant to type I and II IFN-induced apoptosis; 4) T-cell activation is compromised in tumor cells lacking JAK1; and 5) exogenous type I IFN can induce regression of JAK1-deficient tumors by activating host non-tumor immune cells (Figure 7).

It has been reported that absence of JAK1 expression induced human lung adenocarcinoma unresponsive to IFN-γ (30). A recent clinical study showed that loss-of-function mutations in JAK1, JAK2, or β2M were related to resistance to PD-1 blockade therapy (31, 33); another study in which candidate components of the IFN signaling pathway such as IFNGR, JAK1, JAK2, STAT1, and IFN regulatory factor 1 were screened found that JAK1/2 deficiency was associated with T-cell-resistant HLA class I-negative lesions (34). The results of the present study demonstrate that JAK1 mutation plays a key role in the resistance to adoptive T-cell transfer, which is consistent with the findings of previous studies examining immune checkpoint blockade resistance (31–34). Thus, IFN signaling plays a critical role in the decreased responsiveness to various immunotherapies.

We also found that loss of JAK1 in melanoma decreased the cytotoxicity of effector T-cells and expression of associated molecules such as TNF-α, granzyme, and perforin. The expression of MHC-I and PD-L1 was also downregulated in the absence of JAK1; the lower level of MHC-I reduced MHC-I-peptide complex formation, thereby suppressing T-cell activation and preventing recognition by cytotoxic T-cells, leading to immune resistance. However, PD-L1—which functions as a braking signal in T-cell activation—was downregulated in the absence of JAK1, which was at odds with the resistance phenotype. Thus, additional studies on the mechanisms of PD-L1, MHC-I, and other IFN-stimulated genes (ISGs) in T-cell resistance are needed to resolve this inconsistency. ISGs, the downstream effectors of IFN signaling, regulate many cellular processes including apoptosis through NF-κB, Bcl2, and Bax (31), and their own negative feedback through SOCS (37); and MHC-I and PD-L1. In this study, we found that JAK1 deficiency inhibited the apoptosis of tumor cells; however, in tumor cells lacking JAK1, the expression of apoptosis signaling molecules and SOCS was unknown despite changes in MHC-I and PD-L1 downstream of ISGs; therefore, the role of these molecules in immune resistance remains to be determined.

The results of this study have several implications for overcoming cancer immunotherapy resistance. Firstly, we established a CRISPR/Cas9-based method of identifying potential targets for overcoming resistance to adoptive T-cell transfer in melanoma that can be easily applied to other cancers to investigate tumor type-specific inhibitory mechanisms. Secondly, we determined that JAK1 is a candidate molecule for inducing resistance to adoptive T-cell transfer; thus, JAK1 and other IFN pathway components can serve as biomarkers for screening patients that are responsive to this therapy. Finally, we observed that exogenous type I IFN can rescue the effects of JAK1 deficiency *in vivo*, suggesting a possible means for overcoming JAK1 mutation-induced resistance in clinic settings. These findings provide new insights into the mechanism of adoptive T-cell therapy resistance and a basis for the development of novel immunotherapy strategies.

## Author Contributions

PH carried out most of the experiments and wrote the paper. QD, LF, HL, XZ, and FL participated in animal experiments. XY supervised the study and commented on the manuscript.

### Conflict of Interest Statement

The authors declare that the research was conducted in the absence of any commercial or financial relationships that could be construed as a potential conflict of interest.
